# Ultrasensitive quantitation of human papillomavirus type 16 E6 oncogene sequences by nested real time PCR

**DOI:** 10.1186/1750-9378-5-9

**Published:** 2010-05-14

**Authors:** Socorro Hernández-Arteaga, Rubén López-Revilla

**Affiliations:** 1 División de Biología Molecular, Instituto Potosino de Investigación Científica y Tecnológica, Camino de la Presa San José 2055, 78216 San Luis Potosí S.L.P., México

## Abstract

**Background:**

We have developed an ultrasensitive method based on conventional PCR preamplification followed by nested amplification through real time PCR (qPCR) in the presence of the DNA intercalating agent EvaGreen.

**Results:**

Amplification mixtures calibrated with a known number of pHV101 copies carrying a 645 base pair (bp)-long insert of the human papillomavirus type 16 (HPV16) E6 oncogene were used to generate the E6-1 amplicon of 645 bp by conventional PCR and then the E6-2 amplicon of 237 bp by nested qPCR. Direct and nested qPCR mixtures for E6-2 amplification corresponding to 2.5 × 10^2^-2.5 × 10^6 ^initial pHV101 copies had threshold cycle (Ct) values in the ranges of 18.7-29.0 and 10.0-25.0, respectively. The Ct of qPCR mixtures prepared with 1/50 volumes of preamplified mixtures containing 50 ng of DNA of the SiHa cell line (derived from an invasive cervical cancer with one HPV16 genome per cell) was 19.9. Thermal fluorescence extinction profiles of E6-2 amplicons generated from pHV101 and SiHa DNA were identical, with a peak at 85.5°C.

**Conclusions:**

Our method based on conventional preamplification for 15 cycles increased 10,750 times the sensitivity of nested qPCR for the quantitation of the E6 viral oncogene and confirmed that the SiHa cell line contains one E6-HPV16 copy per cell.

## Background

Invasive cervical cancer develops in women with persistent infection by high-risk human papillomavirus (HR-HPV), among which HPV16 and HPV18 are the most frequent types [1], HPV16 being the most prevalent and associated with nearly half of the invasive cervical cancer cases in the world [2,3].

Molecular methods to quantify the number of HPV16 genome copies serve to determine the viral load and the progression of HPV16 neoplastic lesions. Hybrid capture [4] or "nested" PCR [5] are used for molecular diagnosis of HPV infection, whereas qPCR is used to assess viral load and integration to the cellular genome [6,7].

Conventional nested PCR is more sensitive than conventional "direct" PCR to detect HPV16 E6 oncogene sequences [8,9] whose quantitation is reproducible, specific and more sensitive in qPCR mixtures containing the DNA intercalating agent EvaGreen [10].

In this work we developed an ultrasensitive method to quantify E6-HPV16 oncogene sequences in two steps: 1) preamplification of the E6-1 (645 bp) sequence through 15 cycles of direct conventional PCR, and 2) amplification of the E6-2 (237 bp) sequence through nested qPCR in the presence of EvaGreen. Preamplification significantly increased the sensitivity and the method confirmed that the SiHa cell line contains only one copy of the HPV16 genome per cell.

## Results

### E6-2 amplification by direct qPCR

**Figure 1:**
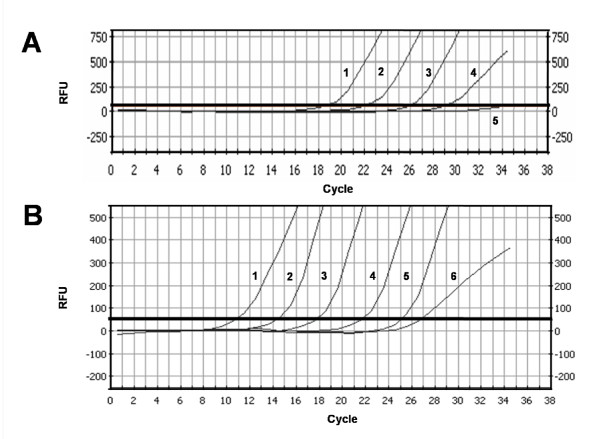
**Family of E6-2 amplification curves obtained by direct and nested qPCR**. RFU, relative fluorescence units. Threshold (thick horizontal line): 50 RFU. (**A**) Family of curves obtained in direct qPCR mixtures with pHV101 dilutions and primers for E6-2. Curve 1, 2.5 × 10^6 ^pHV101 copies. Curve 2, 2.5 × 10^5 ^copies. Curve 3, 2.5 × 10^4 ^copies. Curve 4, 2.5 × 10^3 ^copies. Curve 5, Blank (without DNA). (**B**) Family of curves obtained by nested qPCR with E6-2 primers and 1/50 volume of mixtures preamplified 15 cycles with pHV101 and E6-1 primers. Curve 1, 2.5 × 10^6 ^pHV101 copies. Curve 2, 2.5 × 10^5 ^copies. Curve 3, 2.5 × 10^4 ^copies. Curve 4, 2.5 × 10^3 ^copies. Curve 5, 2.5 × 10^2 ^copies. Curve 6, blank (without DNA).

E6-2 amplification from pHV101 in direct qPCR mixtures containing the pU1M/pU2R primer pair and 2.5 × 10^2 ^to 2.5 × 10^6 ^pHV101 copies in the presence of EvaGreen yielded a consistent family of type curves with Ct values between 18.70 and 29.00, ΔCt values in the 3.1-3.8 range, and lack of fluorescence increase in mixtures devoid of DNA. These experiments indicated that at least 2,500 copies of the E6 oncogene can be quantified by direct qPCR (Fig. 1A).

### Preamplification and nested qPCR

To increase qPCR sensitivity E6-1 was first "preamplified" in conventional PCR mixtures and E6-2 was then "amplified" in nested qPCR mixtures containing EvaGreen, the LCRS/E7AS primer pair, and 1/50 volume of the corresponding preamplified mixtures.

The families of type curves required to calibrate the number of E6 copies and to quantify them in problem samples could be generated from nested qPCR mixtures derived from two types of preamplification mixtures: a) "complete" mixtures, prepared with serial logarithmic dilutions of the pHV101 template molecules before preamplification, and b) "minimum" mixtures, prepared with serial logarithmic dilutions from a single preamplified mixture containing the maximum number of template molecules used in complete mixtures.

Since labor, time and the amount of reagents are significantly reduced using minimum mixtures, we compared the results from nested qPCR mixtures prepared with 1/50 volume samples from preamplified mixtures which contained pHV101 molecules in the range of 2.5 × 10^2^-to 2.5 × 10^6 ^copies with those prepared using serial logarithmic dilutions from a single mixture containing 2.5 × 10^6 ^pHV101 molecules that had been preamplified. We also added 50 ng of "carrier" normal human blood DNA to both complete and minimum positive preamplification mixtures (same amount as SiHa DNA in problem mixtures) and found that all of them generate equivalent nested qPCR amplification results.

Typical Ct values of nested qPCR: 10.80 for 2.5 × 10^6^, 14.35 for 2.5 × 10^5^, 17.71 for 2.5 × 10^4^, 21.62 for 2.5 × 10^3 ^and 25.02 for 2.5 × 10^2 ^initial pHV101 copies in the preamplification mixtures (Table 1) were consistent (ΔCt range: 3.3-3.9) and reproducible (R^2 ^= 1.000), whereas the negative control mixture devoid of template DNA had a Ct = 27 (Fig. 1B).

**Table 1:** Ct values in direct and nested qPCR E6-2 amplicon mixtures with a variable number of pHV101 copies

pHV101 copies	Ct		ΔCt
		
	Direct qPCR	Nested qPCR	
2.5 × 10^2^	---	25.02	---

2.5 × 10^3^	29.00	21.62	7.40

2.5 × 10^4^	25.90	17.71	8.10

2.5 × 10^5^	22.10	14.35	7.75

2.5 × 10^6^	18.70	10.80	7.90

The average decrease in Ct values of nested qPCR resulting from preamplification for 15 cycles was 7.78 (Table 1), corresponding to a 215-fold sensitivity increase. Multiplying this factor by the 50-fold dilution factor of preamplified samples in nested reactions implies that under the conditions used the overall sensitivity of nested qPCR increased 10,750 times.

### E6 copies per SiHa cell

The sensitivity and reliability of the nested qPCR method was tested by determining the HPV16 genome load through quantitation of E6 sequences present in the SiHa line, known to contain only one copy of the HPV16 genome per cell [11].

The following PCR mixtures containing the LCRS/E7AS primer pair and the DNA templates indicated were incubated for 15 cycles under conditions to generate ("preamplify") the E6-1 amplicon: a) positive controls with 2.5 × 10^6^-2.5 × 10^2 ^copies of pHV101 and 50 ng of "carrier" normal human blood DNA; b) negative controls with 50 ng of "carrier" DNA; c) problem samples with 50 ng of SiHa DNA; and d) blanks, without DNA. Nested qPCR mixtures containing EvaGreen, the pU1M/pU2R primer pair and 1/50 volume from each preamplified mixture were incubated for 35 cycles.

**Figure 2:**
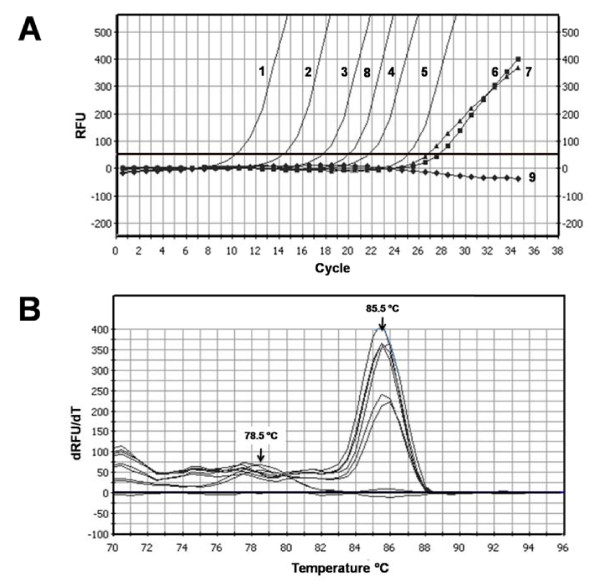
**Thermal denaturation (fluorescence extinction) profiles of the E6-2 amplicons generated from pHV101 and SiHa DNA**. RFU, relative fluorescence units. (**A**) Family of curves obtained with serially diluted pHV101 or 50 ng of SiHa DNA. Threshold (thick horizontal line): 50 RFU. Curve 1, 2.5 × 10^6 ^pHV101 copies. Curve 2, 2.5 × 10^5 ^pHV101 copies. Curve 3, 2.5 × 10^4 ^pHV101 copies. Curve 4, 2.5 × 10^3 ^pHV101 copies. Curve 5, 2.5 × 10^2^pHV101 copies. Curve 6, Blank (without DNA). Curve 7, normal human blood DNA. Curve 8, 50 ng of SiHa cell DNA with the pU1M/pU1R primer pair. Curve 9, 50 ng of SiHa cell DNA with the pU1M forward primer only. (**B**) Thermal fluorescence extinction profiles of nested qPCR products. Profiles of pHV101 and SiHa DNA mixtures with both primers (peaks at 85.5°C), mixtures without DNA or with normal human blood DNA with both primers (peaks at 78.5°C), and SiHa DNA with the pU1M primer only (nearly horizontal line close to the baseline).

In nested qPCR mixtures for E6-2 containing 1/50 volumes of serial logarithmic dilutions from preamplified mixtures with 2.5 × 10^6 ^to 2.5 × 10^2 ^pHV101 molecules the Ct values ranged from 10.8 to 25.0 cycles (R^2 ^= 1.000; slope = -3.57), and the ΔCt values were consistent (range: 3.3-3.9). The Ct values of nested qPCR mixtures prepared without preamplified samples were 27.0 and 28.0, whereas the basal fluorescence decreased slightly in preamplification mixtures containing SiHa DNA with the pU1M primer only (Fig. 2A).

The family of type curves obtained from nested qPCR mixtures for E6-2 amplification was consistent with the initial pHV101 copy number. On the other hand, the Ct value (19.9) and E6 copy number obtained from a typical preamplification mixture containing 50 ng of SiHa cell DNA (equivalent to 6.05 × 10^3 ^HPV16 genome copies) had the magnitudes expected (i.e., one E6 copy per 7.1 pg of DNA, equivalent to the genomic mass of a human diploid cell) (Fig. 2A).

The thermal denaturation ("fluorescence extinction") profiles of nested qPCR products generated from pHV101 and SiHa DNA were identical, with a major peak around 85.5°C whose magnitude was proportional to the initial number of pHV101 copies, and clearly different from the secondary peak at 78.5°C which appeared in the negative controls containing just the forward primer of the second pair (Fig. 2B).

## Discussion

Our group has already confirmed that detection of HPV infection is more sensitive by nested conventional PCR through the successive synthesis of the E6-1 and E6-2 amplicons used in this work [9], and that quantitation of E6 oncogene sequences by qPCR in the presence of EvaGreen is reproducible and specific [10].

The PCR mixtures required to calibrate and quantify the number of E6 copies present in problem samples were prepared using serial logarithmic dilutions of pHV101, a pGEM construct containing a 645 bp-insert whose sequence corresponds to the E6-HPV16 ORF [10].

To increase amplification sensitivity, E6-1 was "preamplified" for 15 conventional PCR cycles and then E6-2 was "amplified" by 35 "nested" qPCR cycles in the presence of normal human DNA using, as source of template, 1/50 volume of either preamplified mixtures prepared with serial logarithmic dilutions of pHV101 or with serial logarithmic dilutions of a single preamplified mixture prepared with the highest number of pHV101 copies. Direct PCR could quantify as little as 2,500 E6-HPV16 molecules, whereas nested qPCR was around 11,000 times more sensitive, a value close to that expected for 15 preamplification cycles which would increase the initial number of E6 template copies by a factor of 2^13 ^= 8,192.

Non-specific fluorescence appears to depend on the interaction of the two E6-2 primers because it increased after cycle 27 in qPCR mixtures prepared with samples from preamplified mixtures containing normal human DNA or HPV16 DNA, but not from those containing SiHa DNA and the E6-2 forward primer only.

Use of the SiHa cell line, originally isolated from an invasive cervical cancer caused by HPV16 and known to contain only one viral genome per cell [11], confirmed the sensitivity and reliability of our nested qPCR method, since preamplified mixtures containing 50 ng of SiHa DNA generated Ct values corresponding to one copy of the E6-HPV16 oncogene per human diploid genome.

The nested qPCR method may also be used in the future to quantify viral load as well as viral and cellular transcripts with high sensitivity and reliability.

## Conclusions

Our method, based on conventional PCR preamplification for 15 cycles increased 10,750 times the sensitivity of nested qPCR for quantitation of the E6 oncogene, and confirmed that the SiHa cell line contains only one E6-HPV16 copy per cell.

## Methods

### Amplification conditions

**Figure 3:**
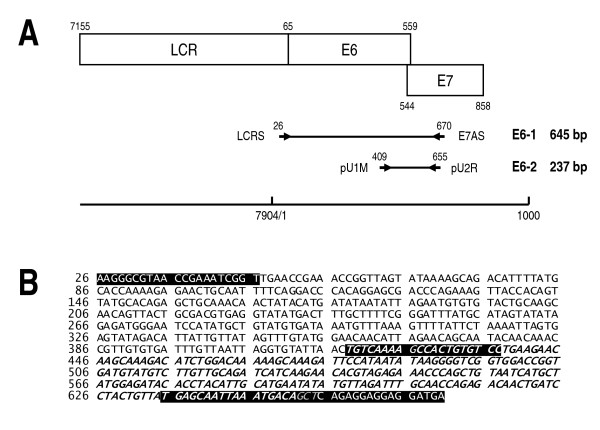
**Sequences of the E6-1 and E6-2 amplicons generated from pHV101**. (**A**) Diagram of the HPV16 long control region (LCR) and the E6 and E7 genes, whose sequences overlap partially. The E6-1 amplicon (645 bp) is generated first by direct PCR with the LCRS/E7AS primer pair and then the E6-2 (237 bp) amplicon is generated by nested PCR with the pU1M/pU2R primer pair. The scale in the lower part of the figure indicates the positions of LCR, the E6 and E7 oncogenes and the E6-1 and E6-2 amplicon sequences on the HPV16 genome. (**B**) The E6-645 insert sequence corresponds to the cloned E6-1 amplicon, spans nucleotides 26-671 (645 bp) of the HPV16 genome and is flanked by the LCRS and E7AS primers (white letters on black background) at the 5' and 3' ends, respectively; it contains 38% GC and is 99% identical to the E6-HPV16 ORF [10]. The fragment corresponding to the E2-237 amplicon (in italics) spans nucleotides 419-656 (237 bp) of the HPV16 genome and is flanked by the pU1M and pU2R primers (white italics on black background), respectively; its sequence contains 41% GC and is identical to the corresponding ORF sequence of the E6-HPV16 oncogene. The three gray italic letters on black background correspond to the overlapping bases of the pU2R and E7AS primers.

E-645, the pHV101 insert sequence spanning the E6-HPV16 open reading frame (ORF), was used as the initial template to generate the E6-1 (645 bp) amplicon through conventional direct PCR and then the E6-2 (237 bp) amplicon through conventional nested PCR (Fig. 3) [10].

E6-1 and E6-2 amplification was maximized in 50 μL conventional PCR mixtures containing 2.5 × 10^6 ^pHV101 copies under the following conditions: 3 mM MgCl_2_, 0.05 mM of each deoxynucleoside triphosphate (dNTP), 0.15 μM of each of the forward and reverse primers (Table 2), 30 U/ml *Taq *polymerase (Invitrogen), and annealing temperature of 57°C.

**Table 2:** Oligonucleotide primers

Pair	Primer	Sequence (5'→3')	Amplicon
1	LCRS (Forward)	AAGGGAGTAACCGAAAACGGT	E6-1 (645 bp)
		
	E7AS (Reverse)	TCATCCTCCTCCTCTGAG	

2	pU1M (Forward)	TGTCAAAAACCGTTGTGTCC	E6-2 (237 bp)
		
	pU2R (Reverse)	GAGCTGTCGCTTAATTGCTC	

PCR products were analyzed by electrophoresis in sodium borate-1.5% agarose gels [12] for 2 h at 90 V, followed by ethidium bromide staining; they were visualized by ultraviolet light transillumination and their digital images recorded in a ChemiDoc EQ photodocumenter (BioRad, Hercules, CA) using the Quantity One (BioRad) software.

### Preamplification and calibration of nested qPCR

Calculation of the number of E6-HPV16 copies is based on the size of pHV101 (3,645 bp) and the average molecular weight of a deoxynucleotide pair (650 Da). We estimated that 1 ng of pHV101 contains 2.5 × 10^8 ^molecules with the formula of Staroscik [13]: number of copies = ((amount in ng) * 6.022 × 10^23^))/((length in bp) * 10^9 ^* 650).

The E6-1 positive control mixtures required to generate the family type curves contained the LCRS/E7AS primer pair, 50 ng of "carrier" normal human DNA and serial logarithmic dilutions of pHV101; they were preamplified for 15 cycles (denaturation at 94°C for 15 sec, annealing at 57°C for 1 min and extension at 72°C for 1 min). Problem preamplification mixtures contained 50 ng of SiHa cell DNA. Negative controls were a blank (i.e., without DNA) and another one with 50 ng of carrier normal human blood DNA.

**Figure 4:**
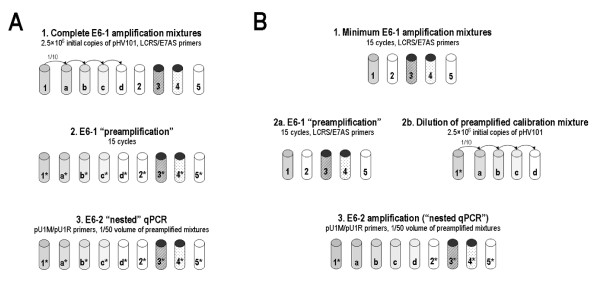
**Complete and minimum E6-1 preamplification mixtures used to perform E6-2 nested qPCR amplification**. (**A**) Complete preamplification series. Successive stages: 1) preparation of E6-1 preamplification mixture containing; 2) "preamplification" by conventional PCR; 3) E6-2 amplification in "nested" qPCR mixtures containing EvaGreen and 1/50 volume of the E6-1 preamplified mixture. Tubes 1, a, b, c and d: positive control preamplification mixtures with serial logarithmic dilutions of pHV101 in the range of 2.5 × 10^6^-2.5 × 10^2 ^molecules per tube. Tube 2: Blank preamplification (without DNA). Tube 3: Problem preamplification (50 ng of SiHa DNA). Tubes 4 and 5: Negative preamplification controls (50 ng "carrier" normal human blood DNA). Asterisks indicate preamplified mixtures. (**B**) Minimum preamplification series. Successive stages: 1) preparation of E6-1 preamplification mixture including only the positive control ("calibration") with the highest pHV101 content; 2a) E6-1 "preamplification" by conventional PCR; 2b) serial logarithmic dilutions of the preamplified calibration mixture; 3) amplification of E6-2 in nested qPCR mixtures containing EvaGreen, the E6-2 primers and 1/50 volume of E6-1 preamplified mixtures. Tube 1: Positive control amplification mixture with 2.5 × 10^6 ^pHV101 molecules. Tube 2: Blank preamplification mixture (without DNA). Tube 3: Problem preamplification mixture (50 ng of SiHa DNA). Tubes 4 and 5: Negative preamplification controls (50 ng "carrier" normal human blood DNA). Tubes a, b, c, and d: serial logarithmic dilutions from the preamplified positive control mixture used to prepare nested qPCR mixtures equivalent to those preamplified with 2.5 × 10^5^-2.5 × 10^2 ^pHV101 molecules. Asterisks of numbered tubes indicate preamplified mixtures.

Positive control preamplification mixtures were prepared either a) as five "complete" PCR mixtures containing serial logarithmic dilutions of pHV101 to attain 2.5 × 10^6^-2.5 × 10^2 ^pHV101 copies/mixture (Fig. 4A), or b) as a single "minimum" PCR mixture containing 2.5 × 10^6 ^pHV101 copies (Fig. 4B); 1/50 volumes from both types of mixtures were added to prepare nested qPCR amplification mixtures.

qPCR mixtures optimized to amplify E6-2 with the pU1M/pU2R primer pair in the presence of EvaGreen (Biotium, Hayward, CA) were incubated with the same thermocycler program. To control nested amplification and to determine the number of E6 copies in problem samples, additional mixtures containing 1/50 volume of preamplified positive, negative and problem mixtures were prepared; in these experiments an additional negative control mixture was used which contained preamplified SiHa DNA and the forward (pU1M) but not the reverse (pU1R) primer (Fig. 4).

## Competing interests

The authors declare that they have no competing interests.

## Authors' contributions

RLR conceived the study and obtained the funds to carry it out. SHA performed the bioinformatics analyses and the molecular studies. Both authors drafted the manuscript.
